# Efficacy of Multimodal Analgesia with Transversus Abdominis Plane Block in Comparison with Intrathecal Morphine and Intravenous Patient-Controlled Analgesia after Robot-Assisted Laparoscopic Partial Nephrectomy

**DOI:** 10.3390/jcm13144014

**Published:** 2024-07-09

**Authors:** Jung-Woo Shim, Dongho Shin, Sung-Hoo Hong, Jaesik Park, Sang Hyun Hong

**Affiliations:** 1 Department of Anesthesiology and Pain Medicine, Seoul St. Mary’s Hospital, College of Medicine, The Catholic University of Korea, Seoul 06591, Republic of Korea; serendip3@catholic.ac.kr (J.-W.S.);; 2 Department of Urology, Seoul St. Mary’s Hospital, College of Medicine, The Catholic University of Korea, Seoul 06591, Republic of Korea

**Keywords:** multimodal analgesia, transversus abdominis plane block, robot-assisted laparoscopic partial nephrectomy, opioid-sparing analgesia

## Abstract

**Background:** Robot-assisted laparoscopic partial nephrectomy (RAPN) for renal tumor treatment provides ergonomic advantages to surgeons and improves surgical outcomes. However, moderate-to-severe pain is unavoidable even after minimally invasive surgery. Despite the growing interest in multimodal analgesia, few studies have directly compared its efficacy with intrathecal morphine, a traditional opioid-based analgesic. **Methods:** We retrospectively investigated the efficacy of multimodal analgesia compared with that of intrathecal analgesia and intravenous patient-controlled analgesia (IV-PCA) in patients who underwent transperitoneal RAPN at our institute between 2020 and 2022. Among the 334 patients who met the inclusion criteria, intrathecal analgesia using morphine 200 µg was performed in 131 patients, and multimodal analgesia, including transversus abdominis plane block and intraoperative infusion of paracetamol 1 g and nefopam 20 mg, was administered to 105 patients. The remaining 98 patients received postoperative IV-PCA alone. **Results:** As the primary outcome, the area under the curve of pain scores over 24 h was significantly lower in the intrathecal analgesia and multimodal analgesia groups than in the IV-PCA group (89 [62–108] vs. 86 [65–115] vs. 108 [87–126] h, *p* < 0.001). Cumulative opioid requirements were also significantly lower in the intrathecal analgesia and multimodal analgesia groups at 24 h after surgery (*p* < 0.001). However, postoperative nausea and vomiting were significantly increased in the intrathecal analgesia group (27.5% vs. 13.3% vs. 13.3%, *p* = 0.005). **Conclusions:** Multimodal analgesia with a transversus abdominis plane block is an efficient analgesic method with fewer adverse effects compared to other analgesic methods. Our findings suggest the efficacy and safety of a multimodal approach for opioid-sparing analgesia after RAPN in the current opioid epidemic.

## 1. Introduction

Surgical approaches to renal tumor resection have evolved from open to laparoscopic [1]. However, the technical challenges in performing laparoscopic procedures have prevented their widespread use [2]. Robot-assisted laparoscopic partial nephrectomy (RAPN) has recently emerged as an alternative to laparoscopic partial nephrectomy [3]. The robot-assisted approach provides surgeons with ergonomic advantages. Moreover, many studies have demonstrated that RAPN improves perioperative outcomes compared to laparoscopic partial nephrectomy, such as lower conversion rates, favorable renal function, and short ischemic time [4,5,6].

However, moderate-to-severe acute pain is unavoidable even after minimally invasive surgery [3]. Poorly controlled acute pain has a series of negative effects on patients after surgery, including slowed functional recovery, decreased patient perception of recovery, increased morbidity, prolonged hospital stay, and progression to persistent pain [7]. Thus, acute pain management remains an issue for enhancing recovery after minimally invasive surgery [8]. Although RAPN has been widely used in clinical settings, the optimal analgesic approach has not yet been thoroughly explored.

In most laparoscopic operations, a multimodal analgesic protocol rather than a single analgesic is recommended for pain control because of the complex nature of pain [9]. Multimodal analgesia should be administered in a surgery-specific way [10,11]. Pain after robot-assisted urological surgery is caused by several surgical stimuli, such as trocar placement, abdominal incisions, pneumoperitoneum, and diaphragmatic stretching [12]. Thus, an ideal analgesic approach for RAPN should manage multiple factors that cause surgical pain with minimal side effects [13].

Traditionally, opioids have been one of the major perioperative analgesics. Among the various opioids and their routes of administration, morphine is administered intrathecally during abdominal surgery [14]. Due to its hydrophilicity, intrathecal morphine exerts prolonged parietal and visceral analgesia in a single dose. However, it accompanies opioid-related adverse effects, including postoperative nausea, pruritus, urinary retention, respiratory depression, and sedation; most of them are unpleasant events, but some are life-threatening [15]. Furthermore, the current opioid epidemic has sparked the implementation of an analgesic strategy based on non-opioid use [10].

Multimodal analgesia is derived from the synergistic effects of non-opioids or interventions with different mechanisms of action, improving pain control and reducing postoperative opioid requirements [10]. The transversus abdominis plane block (TAPB) is an essential component of the multimodal analgesic protocol [16]. TAPB produces analgesia of the anterolateral abdominal wall, avoiding neuraxial analgesia and its related risks.

The co-administration of non-opioids with TAPB has been studied for its effects on patient recovery and pain relief after laparoscopic surgery [17,18,19]. Intravenous (IV) paracetamol is a perioperative medication that is recommended for enhanced recovery because of its opioid-sparing effects [10,13,20]. Additionally, various combinations of nefopam analgesia have been suggested to play a role in multimodal analgesia [21].

To date, few reports have explored the efficacy of multimodal analgesia, including TAPB and non-opioids, compared to intrathecal morphine in RAPN. We retrospectively investigated clinical data from our institute to compare their impact on pain control and recovery after surgery.

## 2. Materials and Methods

This single-center retrospective cohort study was conducted at Seoul St. Mary’s Hospital, a tertiary academic teaching hospital. This study was approved by the hospital’s institutional review board and ethics committee on 21 July 2023 (approval number: KC23RISI0525). Informed consent was not required because of the retrospective nature of the study.

This study was performed through a comprehensive review of the clinical data of patients who underwent RAPN at the Department of Urology of our institute between January 2020 and December 2022. The inclusion criteria for the study patients are as follows: (1) transperitoneal RAPN for renal tumors from January 2020 to December 2022, (2) patients aged ≥19 years and ≤75 years, and (3) patients with American Society of Anesthesiologists Physical Status (ASA-PS) I–II. The exclusion criteria were as follows: (1) open conversion, (2) combined operation, and (3) re-operation until postoperative day (POD) 1.

General anesthesia was induced using IV propofol 1.5–2 mg/kg and rocuronium 0.8–1 mg/kg, followed by endotracheal intubation. Inhaled 5–6% desflurane and IV remifentanil infusion at a rate of 0.01–0.1 µg/kg/min were given to maintain the appropriate depth of anesthesia at the discretion of attending anesthesiologists. Multiple monitoring devices, such as non-invasive blood pressure measurement, pulse oximetry, electrocardiography, bispectral index assessment, esophageal temperature, and end-tidal carbon dioxide level monitoring, were routinely used. Balanced salt solutions were administered at a rate of 2–4 mL/kg/h, with additional replacement for blood loss. Persistent intraoperative hypotension, defined as systolic blood pressure < 80 mmHg, was treated with ephedrine 4 mg unless hypovolemia was suspected. At the end of the surgery, sugammadex 4 mg/kg was injected for neuromuscular blockade reversal. Patients were extubated under ventilation with 100% oxygen when they met the eligibility criteria.

At our institute, all patients were provided IV patient-controlled analgesia (PCA) and rescue opioids after RAPN. Fentanyl-based PCA was infused after arrival at the postanesthetic care unit (PACU). According to the pain management protocols for RAPN at our institute, the fentanyl dose was set at 10 µg bolus injection and 10 µg/h basal infusion, with a lockout interval of 10 min and no loading dose. The patients were instructed to use a PCA bolus when moderate postoperative pain with intensity ≥ 4 on a numeric rating scale (NRS) occurred. The rescue analgesics were administered when moderate pain continued despite the use of PCA boluses or acute severe pain (NRS score ≥ 7) occurred. As rescues, a bolus of fentanyl 50 µg (Daehan Pharma. Co., Seoul, Republic of Korea) was administered in the PACU, and 50 mg of tramadol (Yuhan Pharma. Co., Seoul, Republic of Korea) was used in the ward. In patients aged > 75 years or with ASA-PS ≥ III, the regimen of PCA and doses of rescue opioids were determined at the discretion of the anesthesiologist presiding the surgery and attending staff in the ward.

Intrathecal analgesia was administered between January 2021 and February 2022. Before anesthesia induction, the patients were placed in the lateral decubitus or sitting position. The attending anesthesiologist performed an intrathecal block using morphine and normal saline (1.2 mL in total). Morphine 200 µg (BCWORLD Pharm. Co., Seoul, Republic of Korea) mixed with normal saline (1 mL) was infused after a clear cerebrospinal fluid was observed when a sterile 25-G Quincke-type spinal needle was inserted between the lumbar vertebrae 3 and 4 or 4 and 5.

Multimodal analgesia was introduced in March 2022 to replace intrathecal analgesia. Preoperatively, TAPB with intraoperative administration of non-opioids comprised a multimodal analgesic protocol. In cases of RAPN using the transperitoneal approach, a unilateral TAPB was performed immediately after anesthesia induction by the attending staff on the operative side. Under ultrasound guidance, a sterile 21-G 8.5 mm needle (Echoplex; Vygon Co., Paris, France) was carefully advanced in-plane in a medial-to-lateral direction at the level of the umbilicus. To ensure the posterior spread of the local anesthetic, the ultrasound probe was placed in a transverse orientation at the midaxillary line. After the needle tip was positioned inside the transversus abdominis plane between the internal oblique and transversus abdominis and negative aspiration was confirmed, 20 mL of 0.25% ropivacaine (Mitsubishi Tanabe Pharm. Co., Osaka, Japan) was injected. Additionally, an intraoperative bundle of analgesics, paracetamol 1 g (Woosung Pharm. Co., Suwon, Gyeonggi-do, Republic of Korea) and nefopam 20 mg (Myungmoon Pharma. Co., Seoul, Republic of Korea), were separately infused over 15 min at the beginning of surgery in all patients.

The patients were divided into three groups according to the analgesic measures they received during each period: IV-PCA alone (IV-PCA group) from January to December 2020, intrathecal morphine and IV-PCA (intrathecal analgesia group) from January 2021 to February 2022, and multimodal analgesia with TAPB and IV-PCA (multimodal analgesia group) from March to December 2022.

Following the induction of general anesthesia, a urinary catheter was routinely inserted. After the patient was placed in the modified lateral decubitus position, the standard surgical procedure for RAPN in the Department of Urology of our institute was performed as follows.

**Figure 1:**
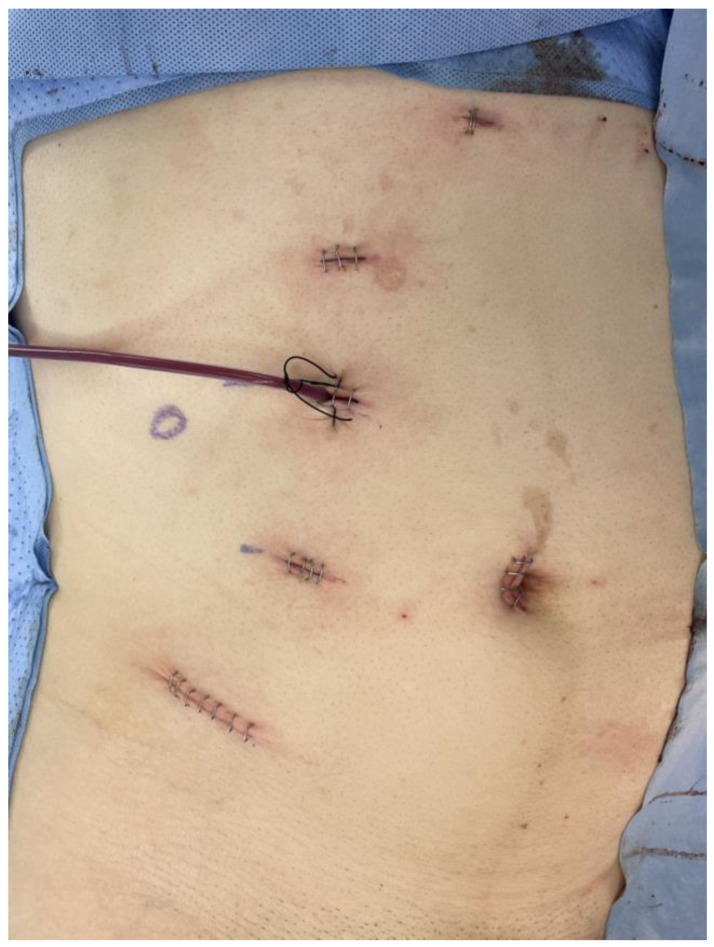
Locations of operative scars in robot-assisted laparoscopic partial nephrectomy using the transperitoneal approach.

The da Vinci Xi Surgical System (Intuitive Surgical Inc., Sunnyvale, CA, USA) was installed according to the manufacturer’s instructions. Using the transperitoneal approach, a 12 mm assistant port was placed through a supra-umbilical incision, and four 8 mm trocars for the robotic arms were placed in a linear configuration after pneumoperitoneum was achieved. For right-sided cases, an additional 5 mm subxiphoid port was placed for liver retraction. Under adequate traction on the kidney, perirenal fat was dissected from the mid-pole to the inferior pole to expose the hilum. After pedicle dissection, IV mannitol 0.5 mg/kg was infused. Renal vessels were clamped using laparoscopic bulldog clamps. Dissection of the tumor mass was carefully performed to achieve a negative surgical margin. The excised kidney was sutured with continuous running sutures using a wound-closure V-Loc device (Medtronic, Minneapolis, MN, USA). Hem-o-lok clips (Teleflex Medical, Research Triangle Park, NC, USA) were placed over the sutures, the sliding-clip renorrhaphy, to apply pressure to the parenchyma and maintain hemostasis. The bulldog clamps were then removed, applying Surgicel (Ethicon Endo-Surgery, Somerville, NJ, USA) and fibrin glue (Tisseel; Baxter Healthcare, Deerfield, IL, USA) around the surgical site for additional bleeding control. The resected specimen was extracted after being placed in a laparoscopic surgical bag inserted through an assistant port. A Gibson incision, extending a robotic port above the anterior superior iliac spine to approximately 3–5 cm, was made to retrieve the specimen (Figure 1). Finally, a Jackson–Pratt drain was placed, and the peritoneum, subcutaneous tissue, and skin were closed layer by layer.

The demographic data included age, sex, body mass index, ASA-PS classification, and medical history. Surgical data included the operator, approach type, operation time, and fluid balance. Information regarding anesthetic and analgesic management was also reviewed. Postoperatively, any complications were checked, and surgery-related complications were assessed using the Clavien–Dindo classification.

For postoperative management, the pain intensity of all patients was rated by attending nurses using the NRS scale. They were assessed upon arrival in the PACU and during the hospital stay, at 3 ± 1, 6 ± 1, 12 ± 1, 18 ± 1, and 24 ± 1 h after surgery, using a Likert scale of 0–10; 0 equals no pain and 10 equals the worst pain the patient had ever suffered. In all assessments, the cumulative doses of PCA and rescue opioids were also recorded.

The primary outcome of this study was the area under the curve (AUC) of the NRS over 24 h postoperatively, calculated using the trapezoidal method, to assess time-weighted acute postoperative pain [22]. The secondary outcomes were NRS pain scores, cumulative opioid requirements, and postoperative complications, including postoperative nausea and vomiting (PONV), pruritus, and hypoxia. The incidences of PONV and pruritus were determined based on the prescriptions of rescue antiemetic agents and chlorpheniramine, respectively, up to 24 h. Postoperative hypoxia was defined as a pulse oximeter value ≤ 93% at discharge from the PACU. The cumulative opioid requirement was expressed as IV morphine equivalents (mg), calculated by summing the doses of PCA consumption and all rescue opioids after surgery.

The normality of the continuous data was assessed using the Shapiro–Wilk test. Continuous variables are reported as medians (interquartile range), while categorial variables are reported as frequencies (percentages). Between-group comparisons were made using the Kruskal–Wallis test, χ^2^ test, or Fisher’s exact test, as appropriate. Post hoc tests for variables showing statistical significance were followed and included the Mann–Whitney test with Bonferroni’s method or the χ^2^ test or Fisher’s exact test with Bonferroni’s method, as appropriate. In addition, the temporal patterns in postoperative pain scores were compared between the groups using a generalized estimating equation analysis. Statistical analyses were performed using the SPSS Statistics software (version 24.0; IBM Corp., Armonk, NY, USA). A *p* value < 0.05 was considered statistically significant, while Bonferroni-adjusted *p* value was used for post hoc tests.

## 3. Results

### 3.1. Comparison of Demographic and Surgical Characteristics among the Three Groups

**Figure 2:**
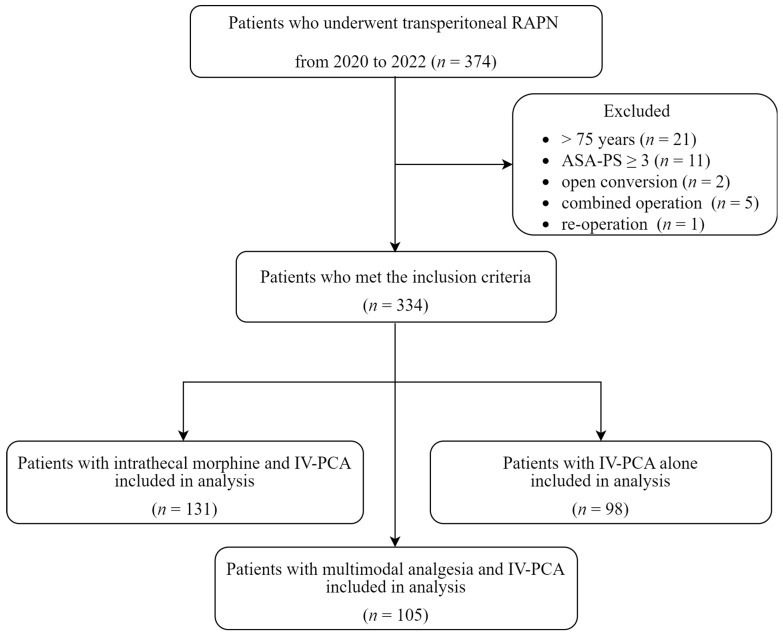
Flowchart of the study. RAPN, robot-assisted laparoscopic partial nephrectomy; ASA-PS, American Society of Anesthesiologists Physical Status; IV-PCA, intravenous patient-controlled analgesia.

From 2020 to 2022, 492 patients underwent RAPN at the Department of Urology at our institute. Among them, 374 underwent transperitoneal RAPN. Out of this cohort, the following 40 patients met the exclusion criteria of our study: 21 patients aged over 75 years old, 11 patients with ASA-PS ≥ 3, 2 patients with open conversion, 5 patients with combined operation, and 1 patient with re-operation until POD 1. Therefore, 334 patients were included in the final analysis. Among the patients, 131 and 105 received intrathecal and multimodal analgesia, respectively, according to the RAPN analgesic protocol at the time of surgery. The remaining 98 patients who underwent transperitoneal RAPN in 2020 before the commencement of intrathecal or multimodal analgesia were allocated to the IV-PCA group. Figure 2 shows a flowchart of the study cohort.

As shown in Table 1, the preoperative characteristics did not differ among the three groups. Intraoperative remifentanil doses were significantly decreased in intrathecal analgesia and multimodal analgesia groups, compared to the IV-PCA group (3.0 [2.2–3.9] vs. 3.0 [2.3–3.6] vs. 3.7 [2.7–4.8], *p* < 0.001). The intrathecal and multimodal analgesia groups did not differ in remifentanil doses. No differences were noted in other demographic or surgical characteristics, and the distribution of operators was similar among the groups.

### 3.2. Comparison of Postoperative Pain Scores and Opioid Requirements among the Three Groups

Table 2 shows that the AUC of NRS pain scores over 24 h after surgery were significantly lower in intrathecal analgesia and multimodal analgesia groups than in the IV-PCA group (89 [62–108] vs. 86 [65–115] vs. 108 [87–126] h, *p* < 0.001). No significant difference in the AUC of the NRS was observed between the intrathecal and multimodal analgesia groups.

Similar to this result, there were between-group differences in NRS pain scores until 12 h after surgery (0 h; 4 [2–5] vs. 4 [1–4] vs. 5 [4–5], *p* < 0.001, 3 ± 1 h; 4 [3–6] vs. 4 [3–6] vs. 5 [4–7], *p* < 0.001, 6 ± 1 h; 4 [3–5] vs. 3 [3–5] vs. 5 [4–6], *p* < 0.001, 12 ± 1 h; 3 [2–4] vs. 3 [2–5] vs. 4 [3–5], *p* = 0.002). Additionally, there were significant differences between intrathecal analgesia and IV-PCA groups in NRS pain scores at 18 ± 1 h and 24 ± 1 h. However, significant differences were not observed between multimodal analgesia and IV-PCA groups in NRS pain scores at 18 ± 1 h and 24 ± 1 h (18 ± 1 h; 3 [2–5] vs. 4 [3–5] vs. 4 (3–5), *p* = 0.001, 24 ± 1 h; 3 [2–5] vs. 3 [3–5] vs. 4 (3–5), *p* = 0.023).

Cumulative opioid requirements were also significantly lower in intrathecal analgesia and multimodal analgesia groups than in the IV-PCA group during 24 h after surgery (3 ± 1 h; 8.8 [5.5–13.1] vs. 9.3 [7.0–13.6] vs. 12.5 [8.9–15.1], *p* < 0.001, 6 ± 1 h; 12.3 [8.0–18.3] vs. 15.3 [9.9–19.7] vs. 18.9 [14.0–25.6], *p* < 0.001, 12 ± 1 h; 19.8 [13.8–27.8] vs. 21.8 [15.1–31.7] vs. 36.6 [25.7–48.4), *p* < 0.001, 18 ± 1 h; 23.8 [16.8–34.4] vs. 30.9 [22.0–43.2] vs. 43.0 [30.7–55.6], *p* < 0.001, 24 ± 1 h; 28.4 [20.1–41.1] vs. 36.7 [25.2–54.8] vs. 49.0 [34.5–65.0], *p* < 0.001). Significant differences between the intrathecal and multimodal analgesia groups were noted at 18 ± 1 and 24 ± 1 h after surgery.

In Table 3, the generalized estimating equation analysis revealed that the pattern of pain scores over 24 h after surgery in the multimodal analgesia group significantly differed from that in the intrathecal analgesia and IV-PCA groups (*p* < 0.001, respectively).

### 3.3. Comparison of Postoperative Complications among the Three Groups

As shown in Table 4, PONV occurred significantly more frequently in the intrathecal analgesia group than in the multimodal analgesia and IV-PCA groups (27.5% vs. 13.3% vs. 13.3%, *p* = 0.005). The incidence of other postoperative complications did not differ among the three groups.

## 4. Discussion

The findings of our study indicated that implementing intrathecal or multimodal analgesia offered significant benefits in pain control in patients who underwent transperitoneal RAPN compared with IV-PCA alone. In particular, patients subjected to multimodal analgesia, including TAPB, experienced similar acute pain relief with less PONV than those subjected to intrathecal analgesia.

Laparoscopic renal surgery induces perioperative pain through the parietal and visceral pathways [23]. Parietal pain arising from port incisions, gas distention, and intraperitoneal dissection causes nociceptive transmission through spinal nerves from T6 to T12. Additionally, intraoperative renal manipulation activates visceral pain pathways through the sympathetic trunk from T10 to L1 [24]. Several analgesic measures, including opioids, non-opioid systemic analgesics, local infiltration, and regional analgesia, have been used to effectively control various stimuli that affect pain after laparoscopic surgery [10,13]. In particular, the growing concern for perioperative opioid use has led to the widespread clinical application of opioid-sparing multimodal analgesia.

As incorporated into an enhanced recovery protocol, combinations of analgesic medications and regional interventions have been developed in the multimodal analgesic strategy [10,13,20]. To enhance pain control with less consumption of opioids, our institute implemented an intraoperative analgesic bundle of IV acetaminophen and nefopam in addition to preoperative TAPB as a multimodal analgesic regimen in transperitoneal RAPN.

The TAPB is one of the most popular abdominal wall blocks. With the development of ultrasound-guided techniques, TAPB has been used clinically for analgesia during abdominal surgeries [25]. TAPB blocks the anterior and lateral cutaneous branches of the thoracolumbar spinal nerves by spreading local anesthetic in the fascial plane between the internal oblique and transverse abdominis muscle [26]. The analgesic efficacy of the TAPB has been demonstrated in laparoscopic and open abdominal surgeries [27,28]. Although several studies have reported that visceral analgesia is not guaranteed [3,29], TAPB is irreplaceable because it is an easily performed and cost-effective regional intervention with proven opioid-sparing benefits after abdominal surgery [30]. Moreover, TAPB is advantageous over neuraxial blocks in avoiding central neuraxis. TAPB is the cornerstone of successful multimodal analgesic regimens developed for abdominal surgeries.

Due to its safety and rare contraindications, except for severe liver illnesses, acetaminophen has been widely used for decades. As a centrally acting analgesic, it directly inhibits N-methyl-D-aspartate receptors and the cyclooxygenase-2 pathway [31]. Many recent multimodal analgesia regimens include acetaminophen to reduce opioid-related complications and postoperative pain [10,13]. Although acetaminophen is not suitable as a single therapy for moderate-to-severe pain [8], it is a versatile and inexpensive analgesic agent in the commercially available IV form, paracetamol. In addition, it has advantages over non-steroidal anti-inflammatory drugs in patients at risk of postoperative bleeding or renal impairments [32]. Considering the issues of new-onset renal impairment associated with warm ischemia-reperfusion injury in RAPN [33], paracetamol was included in our multimodal analgesic protocol.

Nefopam is derived from the nonsedative benzoxazocine [34]. Its mechanism of action depends on the decreased activation of postsynaptic glutamatergic receptors, including N-methyl-D-aspartate receptors. Due to its non-opioid and non-steroidal properties, nefopam is not associated with side effects that limit its usefulness, such as respiratory depression, sedation, tolerance, and gastrointestinal or hepatic injuries [21]. In addition to safety, a synergistic interaction between nefopam and paracetamol has been observed after abdominal surgery. Despite the limited data, nefopam has emerged as a good candidate for inclusion in multimodal analgesia because of its unique properties.

In this study, multimodal analgesia, including preoperative TAPB and intraoperative infusion of paracetamol and nefopam, significantly decreased intraoperative remifentanil consumption as well as pain scores and opioid requirements during the acute postoperative period, compared to IV-PCA alone. Although further research is required, our results show the potential of multimodal analgesia with TAPB as an ideal regimen for perioperative opioid-sparing analgesia. These findings were supported by previous prospective studies in which analgesic benefits were induced by combining abdominal wall block with non-opioids [17,19].

Interestingly, although the AUC of the NRS pain scores over 24 h was comparable between the intrathecal analgesia and multimodal analgesia groups, between-group differences in the temporal pattern of pain scores were shown in the generalized estimating equation analysis. These findings were probably a result of the differences in the effective durations of analgesic measures; the effects of intrathecal morphine usually last more than 24 h after surgery [15], while analgesic durations of paracetamol and nefopam were reported to be 4–6 h [31,35]. Whether TAPB produces prolonged analgesia beyond 12 h remains controversial [36]. The continuous infusion of local anesthetics by placing the catheters in the transversus abdominis plane and the postoperative administration of non-opioids with an around-the-clock regimen will prolong the effects of multimodal analgesic modalities [20].

The following limitations should be noted when interpreting our results: First, this study is retrospective. The study data relied on the accuracy of the electronic medical records from a single institute. Second, there is a possibility that postoperative opioid requirements in the intrathecal analgesia group were underestimated because PONV, which is increased by intrathecal analgesia, might have limited the use of rescue opioids in the ward. Third, despite ultrasound guidance, it was unclear whether all TAPBs achieved a sufficient degree of sensory block to cover the surgical scars, including the Gibson incision to retract the specimen. Fourth, the incidences of PONV and pruritus were determined based on the prescriptions for rescue treatment. Thus, evaluation of mild complications not requiring treatment was limited in our study. Finally, the optimal combinations of multimodal analgesic approaches were not examined because multimodal analgesia was provided according to the protocol during the study period. Further prospective studies are warranted to overcome these limitations.

## 5. Conclusions

Multimodal analgesia, including TAPB, paracetamol, and nefopam infusions, provided acute pain control comparable to that of intrathecal analgesia with fewer PONV occurrences in patients who underwent transperitoneal RAPN. Our findings suggest the efficacy and safety of a multimodal approach for opioid-sparing analgesia in the current opioid epidemic. The ideal regimen for prolonging analgesic duration should be explored in future studies.

## Figures and Tables

**Table 1:** Comparison of demographic and surgical characteristics among the three groups.

Group	Intrathecal Analgesia Group	Multimodal Analgesia Group	IV-PCA Group	*p* Value	Post Hoc Comparisons	*p* Value
	n = 131	n = 105	n = 98			
Demographic characteristics						
Age (years)	55 (44–62)	54 (45–62)	56 (46–64)	0.597		
Male sex	78 (59.5%)	58 (55.2%)	67 (68.4%)	0.149		
Body mass index (kg/m^2^)	24.1 (22.1–26.9)	25.1 (22.9–27.0)	24.8 (22.6–27.6)	0.224		
ASA-PS				0.378		
I	43 (32.8%)	26 (24.8%)	27 (27.6%)			
II	88 (67.2%)	79 (75.2%)	71 (72.4%)			
Hypertension	48 (36.6%)	41 (39.0%)	40 (40.8%)	0.809		
Diabetes mellitus	28 (21.4%)	19 (18.1%)	19 (19.4%)	0.816		
Prior abdominal surgery	41 (31.3%)	24 (22.9%)	30 (30.6%)	0.307		
Laboratory variables						
White blood cell count (×109/L)	6.1 (5.1–7.2)	6.3 (5.3–7.7)	6.4 (5.5–8.0)	0.229		
Hemoglobin (g/dL)	14.2 (12.9–15.3)	14.0 (12.9–15.0)	14.2 (13.1–15.3)	0.803		
Platelet count (×109/L)	254 (219–308)	251 (227–288)	246 (207–292)	0.178		
Creatinine (mg/dL)	0.8 (0.7–1.0)	0.8 (0.7–0.9)	0.8 (0.7–0.9)	0.660		
Albumin (g/dL)	4.7 (4.4–4.8)	4.6 (4.5–4.8)	4.6 (4.3–4.8)	0.137		
INR	1.0 (1.0–1.0)	1.0 (0.9–1.0)	1.0 (1.0–1.0)	0.214		
Surgical characteristics						
Operator, A/B/C	99/24/8	81/17/7	75/11/12	0.302		
Operation time (min)	150 (130–175)	145 (129–170)	148 (120–175)	0.415		
Remifentanil dose (μg/kg/h)	3.0 (2.2–3.9)	3.0 (2.3–3.6)	3.7 (2.7–4.8)	<0.001	Intrathecal analgesia-multimodal analgesia	>0.597
					Intrathecal analgesia-IV-PCA	<0.001
					Multimodal analgesia-IV-PCA	<0.001
Fluid infusion rate (mL/kg/h)	3.7 (2.6–5.3)	3.8 (2.8–4.8)	3.8 (2.7–5.7)	0.741		
Urine output rate (mL/kg/h)	0.7 (0.5–1.3)	0.7 (0.5–1.1)	0.9 (0.5–1.4)	0.219		
Estimated blood loss rate (mL/kg/h)	0.6 (0.4–1.0)	0.6 (0.4–1.1)	0.6 (0.4–1.1)	0.970		

Abbreviations: ASA-PS, American Society of Anesthesiologists Physical Status; INR, international normalized ratio; IV-PCA, intravenous patient-controlled analgesia. Note: Values are expressed as median (interquartile) and number (proportion). A post hoc test using the Bonferroni correction was used to determine the differences between groups.

**Table 2:** Comparison of postoperative pain scores and opioid requirements among the three groups.

Group	Intrathecal Analgesia Group	Multimodal Analgesia Group	IV-PCA Group	*p* Value	Post Hoc Comparisons	*p* Value
	n = 131	n = 105	n = 98			
NRS pain score						
AUC of NRS over 24 h (h)	89 (62–108)	86 (65–115)	108 (87–126)	<0.001	Intrathecal analgesia-multimodal analgesia	0.775
					Intrathecal analgesia-IV-PCA	<0.001
					Multimodal analgesia-IV-PCA	0.001
^†^ At 0 h after surgery	4 (2–5)	4 (1–4)	5 (4–5)	<0.001	Intrathecal analgesia-multimodal analgesia	0.136
					Intrathecal analgesia-IV-PCA	<0.001
					Multimodal analgesia-IV-PCA	<0.001
At 3 ± 1 h after surgery	4 (3–6)	4 (3–6)	5 (4–7)	<0.001	Intrathecal analgesia-multimodal analgesia	0.056
					Intrathecal analgesia-IV-PCA	0.003
					Multimodal analgesia-IV-PCA	<0.001
At 6 ± 1 h after surgery	4 (3–5)	3 (3–5)	5 (4–6)	<0.001	Intrathecal analgesia-multimodal analgesia	0.355
					Intrathecal analgesia-IV-PCA	0.001
					Multimodal analgesia-IV-PCA	<0.001
At 12 ± 1 h after surgery	3 (2–4)	3 (2–5)	4 (3–5)	0.002	Intrathecal analgesia-multimodal analgesia	0.595
					Intrathecal analgesia-IV-PCA	<0.001
					Multimodal analgesia-IV-PCA	0.010
At 18 ± 1 h after surgery	3 (2–5)	4 (3–5)	4 (3–5)	0.001	Intrathecal analgesia-multimodal analgesia	0.033
					Intrathecal analgesia-IV-PCA	<0.001
					Multimodal analgesia-IV-PCA	0.125
At 24 ± 1 h after surgery	3 (2–5)	3 (3–5)	4 (3–5)	0.023	Intrathecal analgesia-multimodal analgesia	0.263
					Intrathecal analgesia-IV-PCA	0.006
					Multimodal analgesia-IV-PCA	0.113
Cumulative opioid requirement (mg, intravenous morphine equivalents)						
At 3 ± 1 h after surgery	8.8 (5.5–13.1)	9.3 (7.0–13.6)	12.5 (8.9–15.1)	<0.001	Intrathecal analgesia-multimodal analgesia	0.212
					Intrathecal analgesia-IV-PCA	<0.001
					Multimodal analgesia-IV-PCA	0.002
At 6 ± 1 h after surgery	12.3 (8.0–18.3)	15.3 (9.9–19.7)	18.9 (14.0–25.6)	<0.001	Intrathecal analgesia-multimodal analgesia	0.032
					Intrathecal analgesia-IV-PCA	<0.001
					Multimodal analgesia-IV-PCA	<0.001
At 12 ± 1 h after surgery	19.8 (13.8–27.8)	21.8 (15.1–31.7)	36.6 (25.7–48.4)	<0.001	Intrathecal analgesia-multimodal analgesia	0.065
					Intrathecal analgesia-IV-PCA	<0.001
					Multimodal analgesia-IV-PCA	<0.001
At 18 ± 1 h after surgery	23.8 (16.8–34.4)	30.9 (22.0–43.2)	43.0 (30.7–55.6)	<0.001	Intrathecal analgesia-multimodal analgesia	<0.001
					Intrathecal analgesia-IV-PCA	<0.001
					Multimodal analgesia-IV-PCA	<0.001
At 24 ± 1 h after surgery	28.4 (20.1–41.1)	36.7 (25.2–54.8)	49.0 (34.5–65.0)	<0.001	Intrathecal analgesia-multimodal analgesia	<0.001
					Intrathecal analgesia-IV-PCA	<0.001
					Multimodal analgesia-IV-PCA	0.002

Abbreviations: IV-PCA, intravenous patient-controlled analgesia; NRS, numerical rating scale; AUC, area under the curve. Note: Values are expressed as median (interquartile) and number (proportion). A post hoc test using the Bonferroni correction was used to determine the differences between groups. ^†^ Pain score measured at arrival in the post-anesthetic care unit.

**Table 3:** Analysis of generalized estimating equations for the temporal patterns in pain scores.

	Estimate	SE	95% Confidence Limits		*p* Value
Intrathecal analgesia vs. multimodal analgesia	−0.190	0.0507	−0.289	−0.091	<0.001
Intrathecal analgesia vs. IV-PCA	0.049	0.0604	−0.070	0.167	0.421
Multimodal analgesia vs. IV-PCA	0.234	0.0628	0.111	0.357	<0.001

Abbreviations: SE, standard error; IV-PCA, intravenous patient-controlled analgesia.

**Table 4:** Comparison of postoperative complications among the three groups.

Group	Intrathecal Analgesia Group	Multimodal Analgesia Group	IV-PCA Group	*p* Value	Post Hoc Comparisons	*p* Value
	n = 131	n = 105	n = 98			
PONV	36 (27.5%)	14 (13.3%)	13 (13.3%)	0.005	Intrathecal analgesia-multimodal analgesia	0.008
					Intrathecal analgesia-IV-PCA	0.009
					Multimodal analgesia-IV-PCA	0.989
Pruritus	16 (12.2%)	5 (4.8%)	6 (6.1%)	0.079		
Hypoxia	2 (1.5%)	4 (3.8%)	1 (1.0%)	0.323		
^‡^ Major complications	2 (1.5%)	3 (2.9%)	0 (0%)	0.246		
Hospital stay	5 (5–6)	5 (5–5)	5 (5–6)	0.122		

Abbreviations: IV-PCA, intravenous patient-controlled analgesia; PONV, postoperative nausea and vomiting. Note: A post hoc test using the Bonferroni correction was used to determine the differences between groups. ^‡^ Major complications were defined as Clavien–Dindo classification ≥ 3.

## Data Availability

The datasets generated and/or analyzed during the current study are not publicly available because due to ethical restrictions, and only de-identified datasets are available from the corresponding author upon reasonable request.
